# The influence of oxygen and methane on nitrogen fixation in subarctic *Sphagnum* mosses

**DOI:** 10.1186/s13568-018-0607-2

**Published:** 2018-05-05

**Authors:** Martine A. R. Kox, Sanni L. Aalto, Timo Penttilä, Katharina F. Ettwig, Mike S. M. Jetten, Maartje A. H. J. van Kessel

**Affiliations:** 1 Department of Microbiology, Radboud University, Nijmegen, The Netherlands; 2 Department of Biological and Environmental Science, University of Jyväskylä, PO Box 35, 40014 Jyväskylä, Finland; 3 Department of Environmental and Biological Sciences, University of Eastern Finland, PO Box 1627, 70211 Kuopio, Finland; 4 Natural Resources Institute Finland, PO Box 2, 00791 Helsinki, Finland

**Keywords:** Diazotrophy, Methane oxidation, Oxygen, Peatland, *Sphagnum* moss, 16S rRNA amplicon sequencing

## Abstract

Biological nitrogen fixation is an important source of bioavailable nitrogen in *Sphagnum* dominated peatlands. *Sphagnum* mosses harbor a diverse microbiome including nitrogen-fixing and methane (CH_4_) oxidizing bacteria. The inhibitory effect of oxygen on microbial nitrogen fixation is documented for many bacteria. However, the role of nitrogen-fixing methanotrophs in nitrogen supply to *Sphagnum* peat mosses is not well explored. Here, we investigated the role of both oxygen and methane on nitrogen fixation in subarctic *Sphagnum* peat mosses. Five species of *Sphagnum* mosses were sampled from two mesotrophic and three oligotrophic sites within the Lakkasuo peatland in Orivesi, central Finland. Mosses were incubated under either ambient or low oxygen conditions in the presence or absence of methane. Stable isotope activity assays revealed considerable nitrogen-fixing and methane-assimilating rates at all sites (1.4 ± 0.2 µmol ^15^N–N_2_ g^−1^ DW day^−1^ and 12.0 ± 1.1 µmol ^13^C–CH_4_ g^−1^ DW day^−1^, respectively). Addition of methane did not stimulate incorporation of ^15^N-nitrogen into biomass, whereas oxygen depletion increased the activity of the nitrogen-fixing community. Analysis of the 16S rRNA genes at the bacterial community level showed a very diverse microbiome that was dominated by *Alphaproteobacteria* in all sites. Bona fide methane-oxidizing taxa were not very abundant (relative abundance less than 0.1%). Based on our results we conclude that methanotrophs did not contribute significantly to nitrogen fixation in the investigated peatlands.

**Electronic supplementary material:**

The online version of this article (10.1186/s13568-018-0607-2) contains supplementary material, which is available to authorized users.

## Introduction

Biological nitrogen (N_2_) fixation is of great importance to the Earth’s biosphere as it is the major natural process to replenish biologically available nitrogen. In nutrient-limited ecosystems, the better competitors are often those that find alternative ways to gain their required nutrients, i.e. by engaging in a symbiosis with other organisms (van der Heijden et al. 2008). Peatlands are nutrient-limited ecosystems that have been studied intensively due to their significant role in the global carbon (C) cycle. Approximately 1/3 of the global terrestrial carbon is stored as dead organic matter in peatlands (Gorham 1991). In *Sphagnum*-dominated peatlands, *Sphagnum* mosses are the ecosystem engineers. They outcompete vascular plant species in various ways (Malmer et al. 2003), but mainly by creating and maintaining acidic (pH 3–5) and waterlogged conditions. In addition, their own biomass is difficult to degrade, which contributes to the slow decomposition and consequential accumulation of dead organic matter (Clymo 1963, 1964; van Breemen 1995). Peat fens that are mesotrophic or oligotrophic receive N from atmospheric deposition and ground water inflow. Compared to mesotrophic fens, oligotrophic fens receive less nutrients, leading to nutrient limitation and lower productivity (Larmola et al. 2014). In both systems, *Sphagnum* mosses minimize nutrient availability for vascular plants by rapid and efficient nutrient uptake (Fritz et al. 2014).

In these nutrient-limited peatlands, *Sphagnum* circumvents N-limitation by engaging in a relationship with N_2_ fixing microorganisms (diazotrophs). Diazotrophs convert atmospheric N_2_ to ammonia (NH_3_). This is a costly process (16 ATP per N_2_ molecule) catalyzed by an oxygen (O_2_) sensitive nitrogenase enzyme (Postgate 1982). In nature, diazotrophs are abundant, diverse and exist as free-living state as well as in symbiosis with plants. Many moss species are known to harbor a diverse diazotrophic community (Leppänen et al. 2013; Vile et al. 2014; Knorr et al. 2014; Kox et al. 2016; Weston et al. 2015). The diazotrophic activity associated with *Sphagnum* supports and explains high concentrations of N in *Sphagnum* biomass (Vile et al. 2014). Although it is evident that the mosses benefit from the N supply by diazotrophs, the benefits for the microorganism are less apparent. Especially, since it was recently postulated that optimal conditions for diazotrophic microorganisms and the moss-host are very different (van den Elzen et al. 2017).

Biological N_2_ fixation activity is most commonly determined using the acetylene reduction assay (Hardy et al. 1968). Initial studies on the *Sphagnum* association with N_2_ fixing partners indicated that mainly cyanobacteria contributed to the incorporation of N in *Sphagnum* biomass (Berg et al. 2012; Lindo et al. 2013). Although the acetylene reduction assay is a sensitive and easy way to determine N_2_ fixing activity, acetylene itself is an irreversible inhibitor of the methane monooxygenase enzyme in methane (CH_4_) oxidizing bacteria (methanotrophs). This prevents methanotrophs from metabolizing carbon, ultimately leading to cell death. Due to the use of acetylene to measure N_2_ fixation rates, the role of diazotrophic methanotrophs may have been underestimated (Leppänen et al. 2013; Vile et al. 2014). As an alternative method to measure N_2_ fixation, ^15^N–nitrogen (^15^N–N_2_) stable isotope incorporation can be used. With this method, Vile et al. (2014) showed a significant contribution of CH_4_ dependent N_2_ fixation to the *Sphagnum* N-pool (Vile et al. 2014). Several *Sphagnum* 16S rRNA gene-based microbiome studies indicated that *Alphaproteobacteria* were the most abundant N_2_ fixing bacteria (Bragina et al. 2012, 2014; Shcherbakov et al. 2013; Warren et al. 2017). In addition, diversity studies based on nitrogenase *nifH* gene also showed that *Alphaproteobacteria* were highly represented (Bragina et al. 2012; Vile et al. 2014; Kox et al. 2016; Warren et al. 2017). Some of these N_2_ fixing *Alphaproteobacteria* were found to be methanotrophs (i.e. type II *Methylosinus* spp., *Methylocystis* spp.) as well.

Methanotrophs associated with *Sphagnum* mosses have been shown to support moss growth by producing carbon dioxide (CO_2_) which is subsequently taken up by *Sphagnum*, especially under CO_2_ limiting conditions (Raghoebarsing et al. 2005; Kip et al. 2010). The methanotrophs are hypothesized to benefit from O_2_ produced by *Sphagnum* and by being protected from predators inside *Sphagnum*’s hyaline cells (Kostka et al. 2016). However, activity measurements of methanotrophic diazotrophs in environmental *Sphagnum* moss samples have yielded contrasting results (Larmola et al. 2014; Leppänen et al. 2014). Due to the high energy demand of N_2_ fixation, a methanotrophic diazotroph requires high methanotrophic activity in order to sustain N_2_ fixation. Therefore, parameters controlling methanotrophy supposedly contribute to the observed variability in the CH_4_ dependent N_2_ fixation (Ho and Bodelier 2015). To this point, the composition of the active N_2_ fixing community associated with *Sphagnum* and factors that affect this activity have been investigated in only a few studies (Ho and Bodelier 2015). The microorganisms involved in biological N_2_ fixation have been identified, but their relative importance and factors controlling their activity remain unresolved (Ho and Bodelier 2015).

The aim of this study was to elucidate the effect of O_2_ and CH_4_ on N_2_ fixation in *Sphagnum* mosses of oligotrophic and mesotrophic subarctic *Sphagnum*-dominated peatlands. *Sphagnum* mosses were sampled from two mesotrophic and three oligotrophic sites (Lakkasuo peatland in Orivesi, Finland). Sphagnum mosses were incubated under either ambient or low oxygen conditions in the presence or absence of ^13^C-labeled CH_4_ and ^15^N-labeled N_2_. Higher N_2_ fixation activity is expected for severe nutrient limited conditions (oligotrophic) compared to mesotrophic conditions. On the other hand, more buffered conditions and higher pH prevailing in mesotrophic peatlands have previously been shown to be beneficial to N_2_ fixation (van den Elzen et al. 2017). We hypothesize that CH_4_ will stimulate N_2_ fixation activity and this activity will be highest in low O_2_ conditions.

## Materials and methods

### Site description and experimental set-up

The study was performed in the subarctic, nutrient limited mire complex Lakkasuo in central Finland (61°47′N 24°18′E; 150 m.a.s.l.). Lakkasuo is a well-studied boreal mire complex, with an annual N input via rainwater of 0.40 g N m^−2^ year^−1^ (Laine 2004). *Sphagnum* mosses were collected in September 2014 from oligotrophic and mesotrophic fens and a rainwater-fed bog within the same peatland basin (Additional file 1: Table S1). The mesotrophic fen site dominated by *Sphagnum subsecundum* (site A) was a wet fen system, whereas mesotrophic fen site B with *Sphagnum obtusum* was drier. The oligotrophic fen site was naturally divided in two patches where *Sphagnum fallax* (site C) and *Sphagnum papillosum* (site D), respectively, were dominant. The rainwater-fed bog site was dominated by *Sphagnum majus* (site E). See Additional file 1: Table S2 for a full overview of the site vegetation index.

### Incubations

To test whether availability of O_2_ and/or CH_4_ would affect microbial N_2_ fixation rates, *Sphagnum* mosses were incubated under two different O_2_ concentrations (ambient and depleted) and with or without ^13^C–CH_4_. N_2_ fixation and CH_4_ oxidation activity were estimated by measuring the incorporation of ^15^N–N_2_ and ^13^C–CH_4_ respectively. To correct for the natural presence of ^15^N and ^13^C in the moss, controls were incubated without any labelled gasses.

Per treatment, moss samples (10 plantlets per bottle, top 3 cm) from each site were incubated in 180 ml plastic Scholl flasks and closed with septum containing screw-caps. Before incubation, the fresh weight (FW) of the mosses was determined. All bottles received 5% ^15^N–N_2_ in the headspace, except for the background controls. ^13^C–CH_4_ treated samples received additionally 5% ^13^C–CH_4_ in the headspace. The headspace of the ambient O_2_ condition consisted of air. Low O_2_ conditions were achieved by replacing (four cycles of vacuum—helium gassing) the headspace with an artificial gas mixture consisting of N_2_ gas (80%), CO_2_ (0.04%) and helium (20%). Oxygen was not added, so that the only source of oxygen was photosynthesis by the mosses. Samples were incubated for 48 h outside under prevailing light and temperature conditions (Jyväskylä, Finland, September 2014).

### Stable isotope incorporation

After the incubation, moss samples were stored at − 20 °C. Next, samples were freeze-dried using an Alpha 1-4LD plus (Martin Christ GmbH, Osterode am Harz, Germany) and stored at − 20 °C. Dried stems from each treatment were pooled and disrupted using a beadbeater at 1500 rpm for 3 min (Microdismembrator U, B. Braun Biotech Int., Meisungen, Germany). To determine the fraction of ^15^N and ^13^C incorporated in plant biomass, approximately 5 and 0.225 mg, respectively, were put into 5 × 8 mm tin cups in duplo. Next, samples were combusted by flash combustion (1800 °C) on a CNS analyzer (EA 1110 Carlo Erba, Thermo Fisher Scientific, Waltham, MA, USA) coupled to an Isotopic Ratio Mass Spectrometer (Finnigan DeltaPlus, Thermo Electro GmbH, Bremen Germany) via an interface (Conflo III, Thermo Electro GmbH, Bremen, Germany). ^15^N and ^13^C content of all mosses before incubation were uniform.

The ^15^N–N_2_ fixation rates were calculated by correcting the δN of the enriched plant material for the natural ^15^N content, after which the corrected ^15^N increase was converted to ^15^N–N_2_ fixation rates (µmol N g^−1^ DW day^−1^). ^13^C–CH_4_ oxidation rates were calculated in a similar fashion as the ^15^N–N_2_ fixation rates. First, the background ^13^C label was subtracted from the measured increase in ^13^C of the biomass. The corrected ^13^C increase was subsequently converted into ^13^C–CH_4_ oxidation rates.

### Porewater composition

At each site pH was measured and pore water samples were taken using rhizons (pore size 2 µm; 5 cm length) at the surface (0 cm), 5, 10 and 15 cm depth. Element concentrations of Al, Ca, Fe, K, Mg, Mn, Na, P, S, Si and Zn in pore water (10 ml, acidified with 1 ml HNO_3_) were analyzed by inductively coupled plasma optical emission spectroscopy (ICP-OES iCAP 6000, Thermo Fisher Scientific, Waltham, MA, USA). Concentrations of NH_4_^+^, NO_3_^−^, PO_4_^3−^ were analyzed colorimetrically with a 3 Auto Analyzer system (Bran and Luebbe GmbH, Norderstedt, Germany) using ammonium molybdate (Henriksen 1965), hydrazine sulfate (Kamphake et al. 1967) or salicylate (Grasshoff and Johannsen 1972). Cl was determined with a Technicon Flame Photometer IV Control (Bran and Luebbe, Norderstedt, Germany).

To determine the concentration of dissolved methane, 3 ml exetainers (Labco, Ceredigion, UK) were prepared with 1 g of NaCl and closed with a septum cap. Next, 1 ml of the porewater collected via rhizons was added to the closed exetainers immediately after sampling. The pressure in the vials was measured and the CH_4_ concentration in the headspace of the exetainer was measured using the GC as described above.

### Olsen-P determination

Digestion of dried and ground *Sphagnum* tissue was performed to obtain the Olsen P concentration as described (van den Elzen et al. 2017). In brief, samples were heated to 120 °C for 45 min in a mixture of 500 µl HNO_3_ (65% w/w) with 200 µl H_2_O_2_ (30% w/w). Next, samples were diluted with demineralized water and measured by inductively-coupled plasma emission spectrometry (IRIS Intrepid II, Thermo Electron corporation, Franklin, MA, USA).

### DNA extraction and 16S rRNA gene amplification

*Sphagnum* mosses for molecular analysis were sampled directly from the field and immediately put in liquid nitrogen. In the laboratory, samples were stored at − 80 °C. DNA extraction was performed on 0.5 g of sample (fresh weight), using the FastDNA SPIN kit for soil (MP Biomedicals, Santa Ana, CA, USA), following manufacturers protocol. Beadbeating was increased to 2 × 1.5 min at 50 Hz using a tissue lyser (LT, Qiagen, Hilden, Germany) DNA yield was assessed using Qubit fluorometric analysis (Thermo Fisher Scientific, Waltham, MA, USA). Barcoded 16S rRNA gene amplicon library was prepared with a 2 step PCR protocol (Berry et al. 2011). Used primers targeted the V3–V4 region of the 16S rRNA gene of most bacteria (341F-785R; Klindworth et al. 2013). The 25 μl PCR reactions for the first PCR contained 12.5 μl Quanta perfecta mix (Quantabio, Beverly, MA, USA), 1 μl of each primer (20 μM), 1 μl DNA (0.5 ng/μl). The PCR program consisted of 25 cycles of 95 °C 1 min, 60 °C, 1 min, 72 °C for 2 min, after which final elongation 10 min 72 °C. The obtained PCR products were checked for purity and size on 1.5% agarose gel. PCR products of 7 parallel reactions were pooled and purified using QIAquick purification kit (Qiagen, Hilden, Germany) following manufacturers protocol. The second PCR was performed to barcode all samples (bcPCR). For bcPCR the gene-specific The same PCR primers (341F and 785R) as for the first PCR reaction were tagged with adapter sequences, specific barcodes and key sequences at the 5′ end, compatible with Ion Torrent sequencing technology (total 60–62 nucleotides per primer). For each sample six nested bcPCR reactions were conducted in parallel, which were combined after product purity and size control. Subsequently, nested PCR products were purified using QIAquick purification kit (Qiagen, Hilden, Germany) following manufacturers protocol.

### Amplicon sequencing

Prior to Ion torrent library construction, concentration and fragment length of samples was determined with a Bioanalyzer 2100 and the High Sensitivity DNA kit (Agilent Technologies, Santa Clara, CA, USA). The libraries were diluted to a final concentration of 26 pM. According to manufacturer’s protocol the library fragments were attached to Ion Sphere particles using the One Touch instrument and Ion PGM Template OT2 400 kit (Life Technologies, Carlsbad, CA, USA). Subsequently, Ion Sphere particles were loaded on an Ion 318 v2 Chip for the first run and an Ion 314 v2 chip for the second run, after which the amplicon libraries were sequenced according to manufacturer’s protocol using the ION PGM Sequencing 400 Kit, using 850 nucleotide flows. Run 1 (318 chip) resulted in 35,790 reads with an average read length of 132 base pairs. Run 2 resulted in 180,870 reads with an average read length of 275 base pairs.

### Sequence analysis

Sequences from both runs were merged resulting in a total of 216,660 reads with an average length of 252 nucleotides. The reads were analysed using Mothur (v1.38; Schloss et al. 2009) and the Mothur 454 SOP (Schloss et al. 2011). First, reads were quality filtered on read length (200–450 bp) allowing for homopolymers (maximum 8), a maximum of 2 differences with the primer sequence and minimum average quality score of 20 over a window of 50 base pairs. Improved reads were aligned to the Silva nr database (release v123) (Quast et al. 2013). Chimera’s were removed using Uchime. Next, reads were classified at bootstrap value of 80%, after which the unwanted and non-target lineages Archaea, Eukaryota, chloroplast, mitochondria and unknown were removed. The final dataset from which OTUs were clustered consisted of 49,975 sequences of which 16,493 were unique, with an average read length of 221 nucleotides (for a detailed overview of quality filtering see Additional file 1: Table S3). OTU’s were clustered using the average clustering algorithm at a cut off of 0.03, followed by singleton removal and resulted in 2001 OTUs. Sequences were deposited in NCBI SRA under project number PRJNA432031.

### Statistical analysis

Data was analyzed using R version 3.4.0 by the R Development Core Team (2017). Normality of the residuals was tested using Shapiro–Wilk’s normality test (stats-package). Homogeneity of variance was tested using Levene’s test (car-package). In the analysis of N_2_ fixation, CH_4_ oxidation activity and pore water composition, the sites were grouped based on their nutritional trophic state, resulting in the levels: mesotrophic and oligotrophic sites. Grouping was only allowed if the difference between species was not significant (p > 0.05), which was tested first using a one-way ANOVA. N_2_ fixation activity data was transformed by square root transformation to permit parametric tests. Next, differences in N_2_ fixation activity under the different O_2_ conditions, presence of ^13^C–CH_4_ and sites (oligotrophic or mesotrophic), were tested using a 3-way ANOVA (stats-package; for sample size (n) see Additional file 1: Table S5). ^13^C–CH_4_ oxidation activity data was normally distributed. Differences in activity between the two oxygen treatments and sites were tested using a 2-way ANOVA (stats-package). All graphs were constructed in R using ggplot2.

For downstream analysis of the community composition the OTU table and taxonomy files generated by Mothur, were imported into R and analyzed using phyloseq (McMurdie and Holmes 2013).

## Results

### Porewater composition

The oligotrophic sites were more acidic compared to the mesotrophic sites and contained less dissolved CH_4_ (see Additional file 1: Figure S1). Concentrations of NH_4_^+^, NO_3_^−^ and PO_4_^−^ were lowest in the rainwater fed bog (not shown) and were present in slightly higher concentrations in both oligotrophic and mesotrophic fens. Moreover, both oligotrophic and mesotrophic fens showed overall higher elemental concentrations of S, Fe Mg, K and Ca, compared to the bog. Olsen P was higher in mosses originating from mesotrophic sites (0.65 ± 0.02 μmol P g^−1^ DW, see Additional file 1: Figure S2) than in oligotrophic sites (0.54 ± 0.01 μmol P g^−1^ DW).

### N_2_ fixation and CH_4_ oxidation activity

**Fig. 1:**
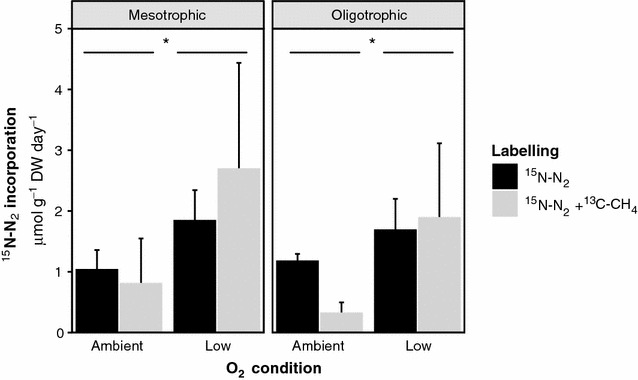
Incorporation of ^15^N–N_2_ (μmol g^−1^ DW day^−1^) in the mesotrophic fens (n = 2) and oligotrophic fens and bog (n = 3) *Sphagnum* mosses incubated with ambient O_2_ conditions (dark grey bars) or low O_2_ conditions (light grey bars), supplemented with 5% ^15^N–N_2_ (dark grey bars) or 5% ^15^N–N_2_ + 5% ^13^C–CH_4_ (light grey bars)

N_2_ fixation rates as measured by ^15^N–N_2_ incorporation were similar in all sites (mesotrophic 1.6 ± 0.4 and oligotrophic 1.5 ± 0.4 μmol ^15^N–N_2_ g^−1^ DW day^−1^). The N_2_ fixation rates were not affected by ^13^C–CH_4_ addition (p > 0.05), but were affected by absence of O_2_ (F_1,16_ = 6.40 p = 0.022; see Fig. 1). Incubation under low O_2_ conditions yielded higher N_2_ fixation activity (2.0 ± 0.5 μmol ^15^N–N_2_ g^−1^ DW day^−1^) compared to incubations at ambient O_2_ conditions (0.8 ± 0.2 μmol ^15^N–N_2_ g^−1^ DW day^−1^).

**Fig. 2:**
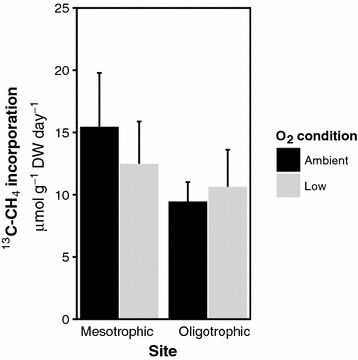
Incorporation of ^13^C–CH_4_ (μmol g^−1^ DW day^−1^) in *Sphagnum* mosses from mesotrophic fens and oligotrophic fens and bog sites, incubated with ambient O_2_ conditions (dark grey bars) or low O_2_ conditions (light grey bars)

CH_4_ oxidation occurred in all incubations, and ^13^C–CH_4_ incorporation was similar (p > 0.05) in all incubations (overall rate 12.0 ± 1.1 µmol ^13^C–CH_4_ g^−1^ DW day^−1^) (Fig. 2).

### Microbial community analysis

**Fig. 3:**
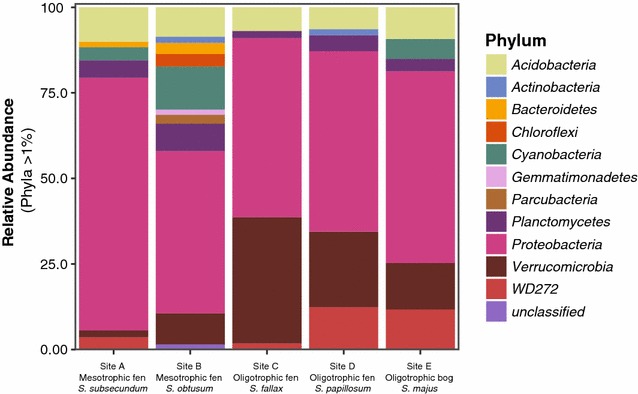
Taxonomic composition (16S rRNA) of the microbial community associated with *Sphagnum* moss from site A–E. Bar charts represent the relative abundance of the different phyla present in each site. Only phyla with a RA > 1% are shown

The bacterial community analysis showed that the *Proteobacteria* were the most dominant phylum in all samples (Fig. 3). In the oligotrophic fen samples *Alphaproteobacteria* were the most abundant class present (> 50% within phylum *Proteobacteria;* see Fig. 3 and Additional file 1: Figure S4). The most abundant families within the *Alphaproteobacteria* are: *Acetobacteraceae* and the *Caulobacteraceae*. The other phyla that made up at least 1% of the microbial community and that appeared to be present in all sites were the *Acidobacteria*, *Verrucomicrobia* and the *Planctomycetes* (Fig. 3). The phylum of *Acidobacteria* had a comparable relative abundance across all sites. In contrast, the *Bacteroidetes and Cyanobacteria* were only present in some sites. Genuine methanotrophic proteobacterial 16S rRNA sequences (type I and type II) were less than 0.1% in relative abundance in all samples.

In the mesotrophic sites, higher microbial diversity was observed (see Additional file 1: Figure S3) compared to the oligotrophic sites. *Verrucomicrobia* were relatively less abundant in the mesotrophic sites compared to the oligotrophic sites, whereas the *Bacteroidetes* (4% of total reads) and *Cyanobacteria* (3% of total reads) were more abundantly found in the mesotrophic sites.

## Discussion

This study aimed to elucidate the effect of CH_4_ and O_2_ availability on N_2_ fixation activity in oligotrophic and mesotrophic *Sphagnum*-dominated peatlands. Biological N_2_ fixation activity was expected to be stimulated by the presence of CH_4_ and the absence of O_2_. For the site effect (oligotrophic vs. mesotrophic), expectations were not so clear-cut, as different confounding factors play a role. Higher availability of N in mesotrophic sites may decrease the demand for biological N_2_ fixation, while higher P content may lead to relative N scarcity. Also, the better buffering and higher pH of mesotrophic sites may be favorable for N_2_ fixation.

In the oligotrophic study sites, however, very low concentrations of the N compounds nitrate and ammonium, typical for oligotrophic conditions, were not found (Additional file 1: Figure S1); they were comparable to concentrations at the mesotrophic sites. Also the N:P relation in porewater was similar, so that no bigger relative N scarcity could be diagnosed for the oligotrophic site. Only in the biomass, slightly higher Olsen P reflected a generally higher availability of P in the mesotrophic site. Taken together, this may explain why N_2_ fixation rates as measured by ^15^N–N_2_ incorporation did not differ between nutritional mesotrophic and oligotrophic trophic states. This is in contrast with recent observations by a study of Van den Elzen et al. (2017), where it was found that N_2_ fixation is stimulated by more buffered conditions and higher phosphorus availability. It is possible that N_2_ fixation rates in our research were affected by other factors such as the relative dry period in which samples were taken or lack of certain trace elements (Vitousek et al. 2013; Warren et al. 2017).

Methane addition lead to significant uptake of ^13^C–CH_4_ derived carbon, but did not affect N_2_ fixation rates compared to controls, indicating that CH_4_ dependent N_2_ fixation is probably not a major contributor to N_2_ fixation in the studied ecosystem. These results are similar to findings of both Leppänen et al. (2014) and Warren et al. (2017). In both studies the addition of methane to batch incubations with Sphagnum did not affect N_2_ fixation activity either. Furthermore, both studies showed much lower CH_4_ oxidation activity than observed in the present study, questioning the possibility of an N_2_-fixing life style of the methanotroph (Ho and Bodelier 2015). Although methanotrophs comprised less than 0.1% of the 16S rRNA, this alone does not exclude them from being a major contributor to N_2_ fixation. Recently Bae et al. (2018) showed that archaea constituting only 0.27% of the microbial community could account for 44% of the N fixed in an Florida peat system. In addition, N_2_ fixation rates may differ over seasons (Lett and Michelsen 2014) and thus the microbial guilds actively performing N_2_ fixation may vary with season. To further confirm which N_2_ fixing microorganisms are active, transcriptomic studies combined with activity assays over different seasons are required in future studies.

In contrast to CH_4_ addition, O_2_ depletion did stimulate N_2_ fixation. While the mosses in the incubation bottles did produce O_2_ as they performed oxygenic photosynthesis during the day, the nevertheless lower O_2_ level resulted in higher N_2_ fixation rates compared to the ambient O_2_ levels, which is in line with our hypothesis. The nitrogenase enzyme that is responsible for N_2_ fixation is irreversibly inhibited by O_2_ in most microorganisms (Vitousek et al. 2002). Our finding indicates that activity of diazotrophs might be strongly controlled by the oxygen concentration, and therefore by depth, position under or above the water level and the current rate of photosynthesis of *Sphagnum*. N_2_ fixation might be higher in the dark than in the light, due to O_2_ release by *Sphagnum* during the light period.

The CH_4_ oxidation rates observed in this study are comparable to CH_4_ oxidation rates measured in other *Sphagnum* mosses (Kip et al. 2010). The CH_4_ oxidation rates were slightly higher in the mesotrophic sites probably due to higher pH. High CH_4_ oxidation activity is essential for methanotrophs in order to fulfil the high energy required for N_2_ fixation (Ho and Bodelier 2015). With these high CH_4_ oxidation rates, energy seems not the limiting factor for CH_4_ dependent N_2_ fixation. Taken together it seems highly unlikely that CH_4_ dependent N_2_ fixation is a major N-supplier to the N-pool of the *Sphagnum* mosses investigated in this study.

### Microbial community composition

The observed microbial community composition of the *Sphagnum* associated microbial community is comparable to the composition found in other studies (Bragina et al. 2012, 2014; Kox et al. 2016) with the *Proteobacteria* as dominant phylum (Bragina et al. 2011, 2014; Putkinen et al. 2014). The potential to perform N_2_ fixation has been reported for many phylogenetic groups (Zehr et al. 2003; Dixon and Kahn 2004; Khadem et al. 2010), of which the *Alphaproteobacteria*, *Cyanobacteria*, *Bacteroidetes* and *Verrucomicrobia* were present in our study. The *Alphaproteobacteria* which were present in all sites, have previously been identified as potentially important N_2_ fixing partners of *Sphagnum* mosses (Bragina et al. 2012; Vile et al. 2014). RT-qPCR and/or transcriptomic sequencing were not performed in this study, therefore we can only speculate which N_2_ fixers were active.

When we compare the different sites, it is apparent that the oligotrophic fens only contain a limited number of cyanobacterial species, whereas the mesotrophic fens do contain more *Cyanobacteria*. Potentially the *Cyanobacteria* may have been affected by the nutrient availability in the peatland, with them becoming more abundant with higher nutrient and productivity levels. The Verrucomicrobial OTU count increased from mesotrophic to the oligotrophic sites, this suggests that species of *Verrucomicrobia* might thrive better under more oligotrophic conditions (Bragina et al. 2015). The high relative abundance of *Verrucomicrobia* present in the peat mosses have been found in earlier studies as well (Putkinen et al. 2014; Bragina et al. 2014). Future isolation of Verrucomicrobial species and physiological studies should reveal their metabolic potential with respect to N_2_ fixation and CH_4_ oxidation.

Up to this point, factors controlling N_2_ fixation in *Sphagnum* mosses have yielded contrasting results. This study has focused on the effect of O_2_ and CH_4_ on N_2_ fixation activity in *Sphagnum* mosses in oligotrophic and mesotrophic subarctic peatlands. Based upon the results we conclude that CH_4_ dependent N_2_ fixation was not a major source of nitrogen for *Sphagnum* mosses at the studied sites. The microbial community associated with *Sphagnum* was dominated by *Proteobacteria* (mainly *Alphaproteobacteria*), which is comparable to other studies. Future studies should combine field and mesocosm studies, with activity assays, community analysis and transcriptomic data to uncover controls of biological nitrogen fixation in *Sphagnum* mosses.

## Additional file

**Additional file 1.** Supplementary tables and figures.
