# Supplementary material for: The influence of oxygen and methane on nitrogen fixation in subarctic Sphagnum mosses
Source: AMB Express. 2018 May 5;8:76. doi: 10.1186/s13568-018-0607-2 (PMC5936483; doi:10.1186/s13568-018-0607-2)
Supplement: Supplementary file 1 — Additional file 1. Supplementary tables and figures. [file 13568_2018_607_MOESM1_ESM.docx]

**Additional file 1**

**Table S1**

Table S1 – Overview of the sampling sites with the *Sphagnum* species and mire types

| **Site** | **Mire type** | **Species** | **GPS location** | **GPS location** |
| --- | --- | --- | --- | --- |
| **A** | Mesotrophic fen | *S. subsecundum* | N61 47.798 | E024 18.513 |
| **B** | Mesotrophic fen | *S. obtusum* | N61 47.658 | E024 18.543 |
| **C** | Oligotrophic fen | *S. fallax* | N61 47.817 | E024 18.515 |
| **D** | Oligotrophic fen | *S. papillosum* | N61 47.817 | E024 18.515 |
| **E** | Ombrotrophic bog | *S. majus* | N61 47.225 | E024 18.590 |

**Table S2**

Table S2 – Vegetation index of plant species present (x) at each site

| Species | Site A | Site B | Site C & D | Site E |
| --- | --- | --- | --- | --- |
| *Andromeda polifolia* |  | x | x | x |
| *Betula nana* |  |  | x |  |
| *Betula pubescens* |  |  | x |  |
| *Carex chordorrhiza* |  | x |  |  |
| *Carex lasiocarpa* |  | x | x |  |
| *Carex limosa* |  | x | x |  |
| *Carex livida* | x |  |  |  |
| *Carex rostrata* |  | x | x |  |
| *Drosera rotundifolia* |  |  | x | x |
| *Equisetum fluviatile* | x | x |  |  |
| *Eriophorum angustifolium* | x |  |  |  |
| *Eriophorum vaginatum* |  |  |  | x |
| *Menyanthes trifoliata* |  |  | x |  |
| *Comarum palustre* |  | x |  |  |
| *Tricophorum cespitosum* | x |  |  | x |
| *Tricophorum alpinum* | x |  |  |  |
| *Sphagnum balticum* |  |  |  | x |
| *Sphagnum fallax* |  | x | x |  |
| *Sphagnum magellanicum* |  | x |  |  |
| *Sphagnum majus* |  |  |  | x |
| *Sphagnum obtusum* |  | x |  |  |
| *Sphagnum papillosum* |  |  | x |  |
| *Sphagnum rubellum* |  |  |  | x |
| *Sphagnum subsecundum* | x |  |  |  |
| *Sphagnum tenellum* |  |  |  | x |
| *Sphagum fuscum* |  |  |  | x |
| *Vaccinium oxycoccos* |  |  | x |  |

**Table S3**

Table S3 - Overview of read abundance during quality filtering

| **Step** | | **# total reads** | **# unique reads** | **Mean read length** |
| --- | --- | --- | --- | --- |
| 1. | Unprocessed reads | 216660 | - | 252 |
| 2. | Quality based trim | 92615 | - | 360 |
| 3. | Unique sequences | 92615 | 84650 | 357 |
| 4. | Align and screen sequences | 84989 | 77668 | 367 |
| 5. | Filter positions in alignment | 84989 | 43353 | 219 |
| 6. | Pre-clustering | 84989 | 21798 | 220 |
| 7. | Chimera removal | 83146 | 20181 | 221 |
| 8. | Unwanted lineage removal | 49975 | 16493 | 221 |

**Table S4**

Table S4 – Operational taxonomic units (OTUs) with a relative abundance of >1%

|  |  | **Similarity** | | | | | | | | **Relative abundance within each site (%)** | | | | |
| --- | --- | --- | --- | --- | --- | --- | --- | --- | --- | --- | --- | --- | --- | --- |
|  |  |  |  |  |  |  |  |  |  | **Site** | **Site** | **Site** | **Site** | **Site** |
| **OTU ID** | **sequence ID** | **Phylum** | **(%)** | **Class** | **(%)** | **Order** | **(%)** | **Family** | **(%)** | **A** | **B** | **C** | **D** | **E** |
| **Otu00006** | UYWTV_00976_01822 | *Acidobacteria* | 100 | *Acidobacteria* | 100 | *Acidobacteriales* | 100 | *Acidobacteriaceae Subgroup1* | 100 | **4.46** | 0.45 | **3.22** | **1.03** | **2.44** |
| **Otu00013** | UYWTV_00057_00665 |  |  |  |  | *Subgroup 3* | 100 | Unknown Family | 90 | **1.27** | 0.19 | 0.85 | 0.52 | **2.72** |
| **Otu00054** | UYWTV_00072_00720 | *Cyanobacteria* | 100 | *Cyanobacteria* | 100 | *Subsection III* | 90 | *Family I* | 90 | 0.00 | **1.39** | 0.01 | 0.00 | 0.00 |
| **Otu00014** | UYWTV_00047_00114 |  |  |  |  | *Subsection IV* | 91 | *Family I* | 90 | 0.38 | 0.65 | 0.01 | 0.00 | **4.41** |
| **Otu00010** | UYWTV_00049_01913 |  |  | *unclassified* | 100 | unclassified | 100 | unclassified | 100 | **1.01** | **6.18** | 0.00 | 0.00 | 0.00 |
| **Otu00038** | UYWTV_00037_00187 | *Proteobacteria* | 100 | *Alphaproteobacteria* | 100 | *Sphingomonadales* | 100 | unclassified | 94 | 0.31 | **1.02** | 0.25 | 0.00 | 0.00 |
| **Otu00056** | UYWTV_00936_02884 |  |  |  |  | *Caulobacterales* | 98 | *Hyphomonadaceae* | 98 | 0.06 | **1.28** | 0.00 | 0.00 | 0.00 |
| **Otu00003** | UYWTV_00045_00435 |  |  |  |  |  | 83 | *Caulobacteraceae* | 81 | **7.15** | 0.15 | **8.51** | **3.25** | 0.83 |
| **Otu00007** | UYWTV_00017_01432 |  |  |  |  |  | 99 | *Caulobacteraceae* | 99 | **4.08** | 0.34 | **1.56** | **2.56** | 0.58 |
| **Otu00011** | UYWTV_00032_01680 |  |  |  |  |  | 100 | *Caulobacteraceae* | 100 | **1.59** | **1.58** | 0.54 | **1.31** | **2.11** |
| **Otu00025** | UYWTV_01732_02391 |  |  |  |  |  | 100 | *Caulobacteraceae* | 100 | **1.08** | 0.31 | 0.24 | 0.83 | 0.05 |
| **Otu00035** | UYWTV_00005_00165 |  |  |  |  | *Rhodospirillales* | 92 | *unclassified* | 85 | 0.03 | 0.01 | 0.03 | 0.04 | **1.81** |
| **Otu00029** | UYWTV_00049_01071 |  |  |  |  |  | 100 | *Acetobacteraceae* | 100 | **1.22** | 0.05 | 0.00 | 0.52 | 0.04 |
| **Otu00002** | UYWTV_00266_01851 |  |  |  |  |  | 100 | *Acetobacteraceae* | 100 | **12.17** | 0.52 | **15.88** | **7.19** | **12.56** |
| **Otu00004** | UYWTV_00046_00237 |  |  |  |  |  | 100 | *Acetobacteraceae* | 100 | **4.79** | 0.94 | **2.32** | **2.32** | **6.38** |
| **Otu00009** | UYWTV_00169_02438 |  |  |  |  |  | 100 | *Acetobacteraceae* | 100 | **1.96** | 0.49 | **1.13** | **1.80** | **3.30** |
| **Otu00017** | UYWTV_00016_01111 |  |  |  |  |  | 100 | *Acetobacteraceae* | 100 | 0.29 | 0.01 | **1.39** | 0.63 | **3.25** |
| **Otu00019** | UYWTV_00570_01437 |  |  |  |  |  | 100 | *Acetobacteraceae* | 100 | 0.20 | 0.03 | **1.82** | **2.71** | **1.27** |
| **Otu00022** | UYWTV_00040_01787 |  |  |  |  |  | 100 | *Acetobacteraceae* | 100 | 0.85 | 0.17 | **1.09** | **0.99** | 0.71 |
| **Otu00023** | UYWTV_00008_01720 |  |  |  |  |  | 100 | *Acetobacteraceae* | 100 | 0.98 | 0.13 | 0.55 | **1.47** | 0.51 |
| **Otu00024** | UYWTV_01158_02656 |  |  |  |  |  | 100 | *Acetobacteraceae* | 100 | **1.17** | 0.02 | 0.48 | 0.83 | 0.20 |
| **Otu00015** | UYWTV_00257_02504 |  |  |  |  |  | 100 | *Acetobacteraceae* | 100 | 0.25 | 0.02 | 0.21 | 0.24 | **4.77** |
| **Otu00008** | UYWTV_00402_01838 |  |  |  |  |  | 100 | *Acetobacteraceae* | 100 | **1.19** | 0.35 | **3.66** | **3.11** | **2.57** |
| **Otu00012** | UYWTV_00044_00027 |  |  |  |  |  | 100 | *Rhodospirillaceae* | 100 | **2.50** | **1.28** | **1.04** | 0.91 | 0.36 |
| **Otu00021** | UYWTV_00004_01950 |  |  |  |  | *Rhizobiales* | 100 | *alphaI_cluster* | 91 | **1.01** | 0.14 | 0.36 | 0.55 | **1.32** |
| **Otu00020** | UYWTV_00075_01330 |  |  |  |  |  | 100 | *Bradyrhizobiaceae* | 100 | **1.46** | 0.89 | 0.21 | 0.24 | 0.72 |
| **Otu00018** | UYWTV_00071_00672 |  |  | *Gammaproteobacteria* | 98 | unclassified | 98 | unclassified | 98 | **1.37** | 0.78 | 0.18 | **1.11** | 0.58 |
| **Otu00001** | UYWTV_00522_01593 | *Verrucomicrobia* | 100 | *Methylacidiphilae* | 100 | *Methylacidiphilales* | 100 | *Methylacidiphilaceae* | 100 | 0.20 | **5.70** | **35.57** | **18.88** | **11.91** |
| **Otu00005** | UYWTV_00030_01293 | *WD272* | 100 | unclassified | 100 | unclassified | 100 | unclassified | 100 | **1.77** | 0.04 | **1.00** | **5.90** | **7.17** |
| **Otu00016** | UYWTV_01504_00774 |  |  |  |  |  |  | unclassified | 100 | 0.94 | 0.05 | 0.27 | **4.87** | 0.73 |
| **Otu00028** | UYWTV_00481_01648 |  |  |  |  |  |  | unclassified | 100 | 0.42 | 0.02 | 0.37 | 0.28 | **1.36** |
| **Otu00047** | UYWTV_00004_00108 |  |  |  |  |  |  | unclassified | 100 | 0.00 | 0.00 | 0.00 | 0.00 | **1.57** |

**Table S5**

Table S5 – Sample sizes for ^15^N_2_ fixation and ^13^CH_4_ oxidation rates

| **O2 condition** | **Nutrient status** | **Incubation** | **n** |
| --- | --- | --- | --- |
| Ambient | Mesotrophic | ^15^N_2_ | 4 |
| Ambient | Mesotrophic | ^15^N_2_ + ^13^CH_4_ | 4 |
| Ambient | Oligotrophic | ^15^N_2_ | 6 |
| Ambient | Oligotrophic | ^15^N_2_ + ^13^CH_4_ | 6 |
| Low | Mesotrophic | ^15^N_2_ | 4 |
| Low | Mesotrophic | ^15^N_2_ + ^13^CH_4_ | 4 |
| Low | Oligotrophic | ^15^N_2_ | 6 |
| Low | Oligotrophic | ^15^N_2_ + ^13^CH_4_ | 6 |

**Figure S1**


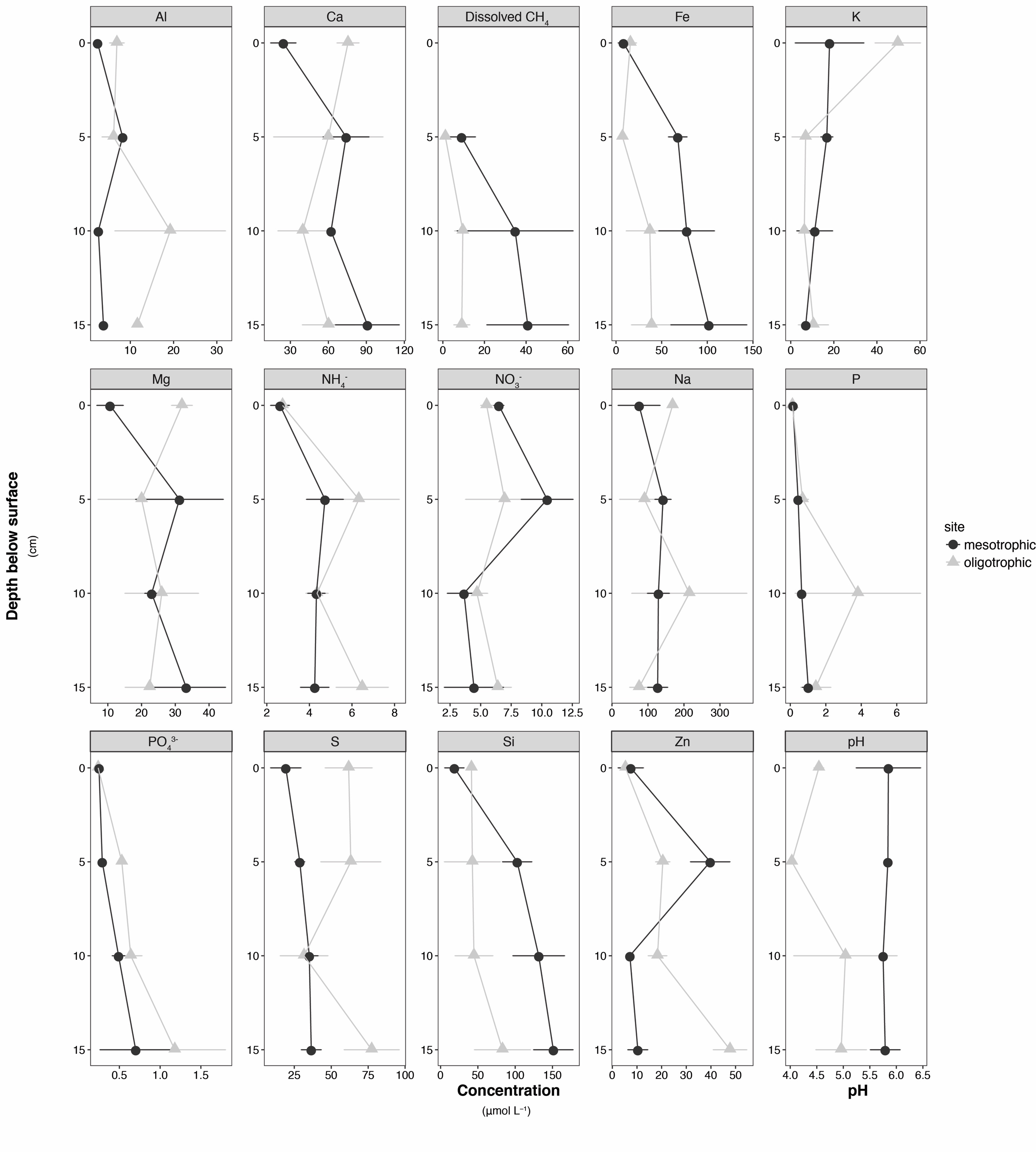


Figure S1 – Overview of concentrations of the different elements, ions and dissolved CH_4_ present in porewater (µmol L^-1^), as well as pH across a depth profile (surface, 0-5 cm, 5-10 cm and 10-15 cm). The mesotrophic (2 fens) and oligotrophic (2 fens, 1 bog) sites were pooled.

**Figure S2**

Figure S2 – Olsen P values (µmol g^-1^ DW) for the mesotrophic and oligotrophic sites.

Mesotrophic

*S. subsecundum*

Mesotrophic

*S. obtusum*

Oligotrophic

*S. papillosum*

Oligotrophic

*S. fallax*

Oligotrophic

*S. majus*

Mesotrophic

*S. subsecundum*

Mesotrophic

*S. obtusum*

Oligotrophic

*S. papillosum*

Oligotrophic

*S. fallax*

Oligotrophic

*S. majus*

**Figure S3**


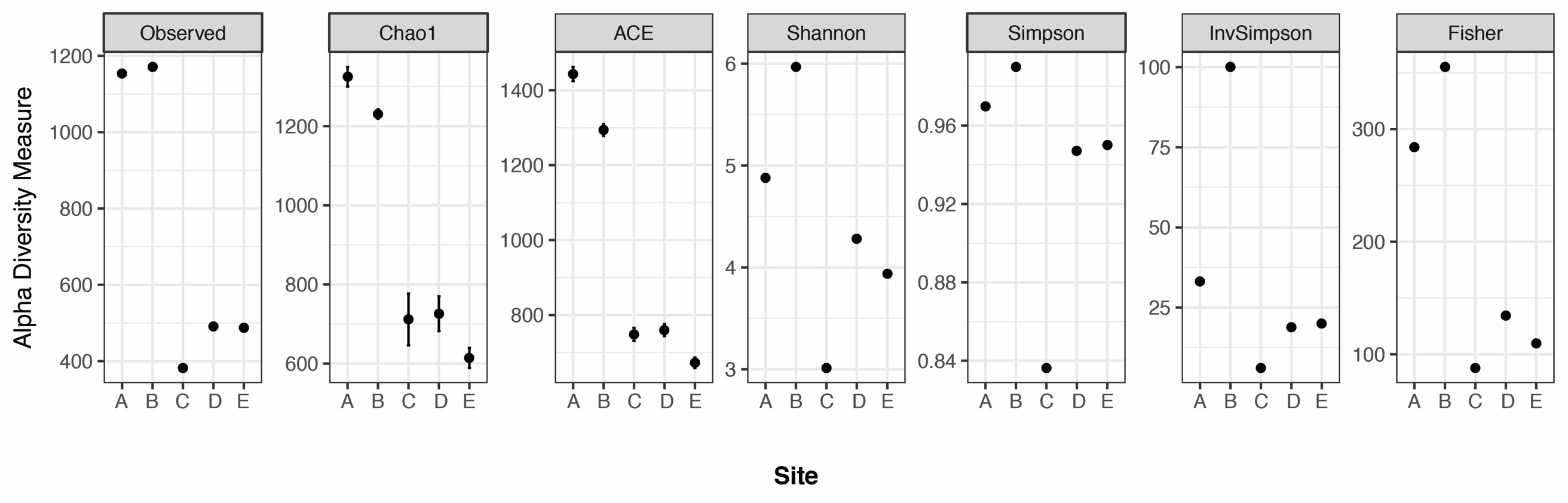


Figure S3 – Alpha diversity indices for the amplicon libraries of each site.

**Figure S4**

**

Figure S4 - Taxonomic composition (16S rRNA) of the microbial community associated with *Sphagnum* moss from site A-E. Bar charts represent the relative abundance of the different classes present in each site. Only classes with a Relative Abundance > 1% are shown.
